# Nucleocapsid Interacts with NPM1 and Protects it from Proteolytic Cleavage, Enhancing Cell Survival, and is Involved in PEDV Growth

**DOI:** 10.1038/srep39700

**Published:** 2017-01-03

**Authors:** Da Shi, Hongyan Shi, Dongbo Sun, Jianfei Chen, Xin Zhang, Xiaobo Wang, Jialin Zhang, Zhaoyang Ji, Jianbo Liu, Liyan Cao, Xiangdong Zhu, Jing Yuan, Hui Dong, Xin Wang, Tiecheng Chang, Ye Liu, Li Feng

**Affiliations:** 1 National Key Laboratory of Veterinary Biotechnology, Harbin Veterinary Research Institute of the Chinese Academy of Agricultural Sciences, No. 678 Haping Road, Xiangfang District, Harbin 150069, P. R. China; 2 College of Animal Science and Veterinary Medicine, Heilongjiang Bayi Agricultural University, No. 2 Xinyang Road, Sartu District, Daqing 163319, P. R. China

## Abstract

Porcine epidemic diarrhea virus (PEDV) replicates in the cytoplasm of infected cells, but its nucleocapsid (N) protein localizes specifically to the nucleolus. The mechanism of nuclear translocation, and whether N protein associates with particular nucleolar components, is unknown. In this study, we confirm that a nucleolar phosphoprotein nucleophosmin (NPM1) interacts and co-localizes with the N protein in the nucleolus. *In vitro* binding studies indicated that aa 148–294 of N and aa 118–188 of NPM1 were required for binding. Interestingly, N protein importation into the nucleolus is independent of the ability of NPM1 to shuttle between the nucleus and the cytoplasm. Furthermore, overexpression of NPM1 promoted PEDV growth, while knockdown of NPM1 suppressed PEDV growth. In addition, binding of N protein to NPM1 protects it from proteolytic degradation by caspase-3, leading to increased cell survival. Taken together, our studies demonstrate a specific interaction of the N protein with the host cell protein NPM1 in the nucleolus. The results suggest potential linkages among viral strategies for the regulation of cell survival activities, possibly through an interaction of N protein with NPM1 which prevents its proteolytic cleavage and enhances cell survival, thus ultimately promoting the replication of PEDV.

Porcine epidemic diarrhea (PED) is an acute, highly contagious, and devastating viral enteric disease with a high mortality rate in sucking pigs. PED was first reported as a clinical entity in England in 1971 and was shown to be distinct from porcine transmissible gastroenteritis (TGE) in 1977^1^. Since then, outbreaks of PED have been reported in many European countries. Currently, PED occurs mainly in Asia, and these outbreaks are more acute and severe than those observed in Europe^2^. In 2010, a large-scale outbreak of PED occurred on swine farms in China and afterwards, in May 2013, this PED virus (PEDV) emerged and spread rapidly in the United States, posing significant economic and public health concerns^3^. The causal agent, PEDV, is a member of the Coronavirinae, which are single-stranded, positive-sense RNA viruses with the largest genome known. They infect humans, other mammals, and birds, usually causing subclinical or respiratory and gastrointestinal diseases. The PEDV genome is composed of a 5′ untranslated region (UTR), at least seven open reading frames (ORF1a, ORF1b, and ORF2 through 6), and a 3′ UTR^4^. ORF1a and ORF1b are located downstream of the 5′ UTR and encode the viral replicase polyproteins 1a and 1b. The remaining ORFs in the 3′ terminal region code for four major structural proteins, the spike (S, 180–220 kDa), envelope (E, 7 kDa), membrane (M, 27–32 kDa), and nucleocapsid (N, 55–58 kDa) proteins, respectively, and ORF3 encodes an accessory protein that is thought to be associated with virulence^5^.

Although there has been much progress in understanding how PEDV causes disease, there remains a paucity of information on the ways in which these pathogens interact with host cells during virus replication and spread. Specifically, we know comparatively little about how individual PEDV proteins interact with host cell factors and how these interactions may lead to porcine disease. The coronavirus N protein is abundantly produced within infected cells. N protein has multiple functions, including as a structural protein that forms complexes with genomic RNA, and plays an important role in enhancing the efficiency of virus transcription and assembly. The identification of host proteins targeted by viral proteins during the infection process provides important insights into the mechanisms of viral protein function. To date, interactions of N protein with numerous host cell proteins have been identified, including hCypA^6^, proteasome subunit p42^7^, Smad3^8^, hnRNP-A1^9^, the chemokine CXCL16^10^, translation elongation factor-1 alpha^11^, cellular pyruvate kinase protein^12^ and 14–3–3^13^. Comparative studies among various coronavirus N proteins could aid the development of novel antiviral therapeutics that target interactions between host cell proteins and the N protein^14^. Manipulation of multiple host cell factors by a relatively small number of viral proteins is critical for virus replication and spread. Given the limited coding capacity of the PEDV genome, its protein products must be multifunctional in order to counter host cell antiviral defenses. Although originally thought to serve purely structural roles, N proteins of coronavirus are emerging as important players at the virus–host interface. Our research group has shown that the PEDV N protein localizes not only in the cytoplasm, but also in the nucleolus in infected cells and cells expressing the N protein alone^15^; however, the factors that determine the nucleolar localization of PEDV N protein and the effect of this localization on virus replication are not clearly understood.

During infection, a number of viral proteins interact with the nucleolus and are able to reorganize nucleolar antigens^16^, with examples from RNA viruses, DNA viruses and retroviruses. These include porcine reproductive and respiratory syndrome virus nucleocapsid protein^17^, hepatitis D virus large-delta antigen^18^, Marek’s disease virus MEQ protein^19^, the adenovirus Iva2 gene product^20^ and V protein^21^, Newcastle disease virus matrix protein^22^, and human immunodeficiency virus type 1 (HIV-1) Rev^23^ and tat^24^. The nucleolus is a highly structured and dynamic nuclear organelle that is involved in the transcription of rRNA and in ribosome biogenesis^25^. It contains many proteins, including nucleophosmin (NPM1/B23), fibrillarin, nucleolin, spectrin, and the ribosomal proteins S5 and L9^26^. In the nucleolus, NPM1 plays a role in centrosome duplication, ribosome biogenesis, intracellular transport, apoptosis and mRNA splicing^27^. NPM1 has been ascribed both growth promoting and tumor suppressive functions^28^,^29^. Loss of NPM1 results in genome instability, which is manifested by aneuploidy, increase in centrosome numbers, and DNA damage checkpoint activation^30^,^31^,^32^. Several different types of cancer cell with elevated levels of NPM1 are more resistant to UV- or hypoxia-induced apoptosis than those with low expression^33^. The ability of NPM1 to suppress apoptosis may play a significant pro-survival role during tumor development^34^.

To date, various studies have focused on the nuclear/nucleolar localization properties of the N protein of coronaviruses^15^,^35^, but information on the interactions of N protein with nucleolar proteins, and their impact on the outcome of PEDV infection, is limited. Here, we show that nucleolus protein NPM1 interacts specifically with the PEDV N protein and positively modulates PEDV growth.

## Results

### Localization of the N protein in PEDV-infected cells

**Figure 1:**
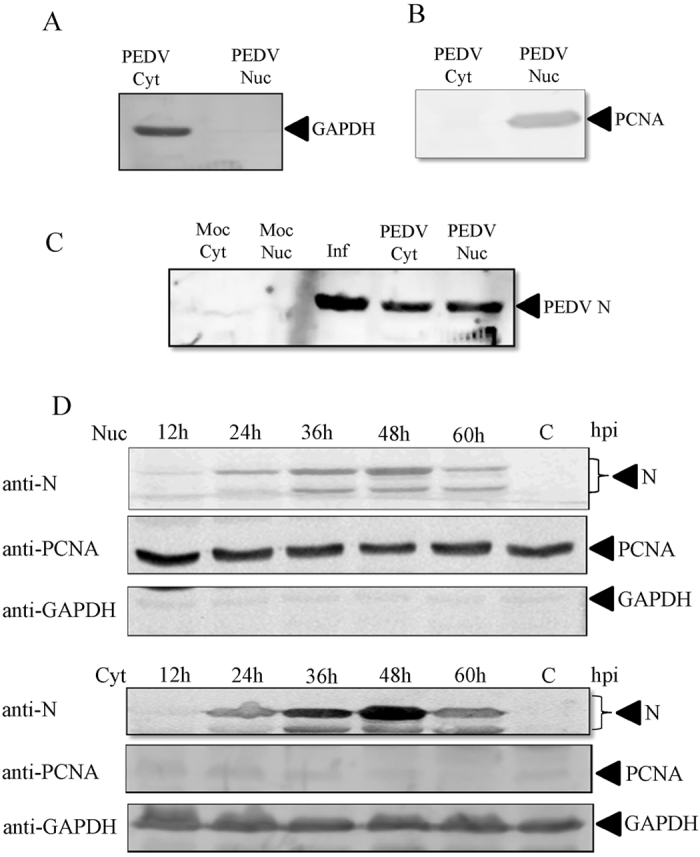
Western blot analysis of N protein in nuclear and cytoplasmic fractions of PEDV-infected Vero E6 cells at 48 h. Western blot analysis of nuclear and cytoplasmic fractions of PEDV-infected Vero E6 cells with an anti-GAPDH mAb (**A**), anti-PCNA mAb (**B**), and anti-N mAb (**C**). (**A,B** and **C**) Nuc, nuclear fraction of PEDV-infected cells; Cyt, cytoplasmic fraction of PEDV-infected cells. (**C**) Moc, mock-infected cells; Inf, PEDV-infected cells. The arrowheads indicate purified bands that are the same sizes as GAPDH (**A**), PCNA (**B**), and N (**C**) proteins. (**D**) Western blot analysis of N protein in nuclear and cytoplasmic fractions of PEDV-infected Vero E6 cells at different times with an anti-GAPDH mAb, anti-PCNA mAb, and anti-N mAb. Nuc, nuclear fraction of PEDV-infected cells; Cyt, cytoplasmic fraction of PEDV-infected cells.

Previous studies using confocal microscopy have shown that N protein is localized in the cytoplasm and nucleolus^15^. In this study, to determine the intracellular distribution of N protein at the protein level, PEDV-infected Vero E6 cells were lysed, separated into nuclear and cytoplasmic fractions, and analyzed by western blotting. As shown in Fig. 1A and B, PCNA protein was detected only in the nuclear fraction, whereas GAPDH was mostly present in the cytoplasmic fraction, confirming the successful separation of the nuclear and cytoplasmic fractions. The N protein was detected in the virus-infected nuclear and cytoplasmic fractions (Fig. 1C, lanes 3, 4 and 5). In addition, multiple bands appeared on the western blots, because the N proteins of coronaviruses are phosphorylated in virus-infected cells (Fig. 1D). These results indicate that N is a protein that shuttles between the nucleus and the cytoplasm, which is consistent with the role of N protein in viral replication.

### N protein interacts and co-localizes with NPM1

**Figure 2:**
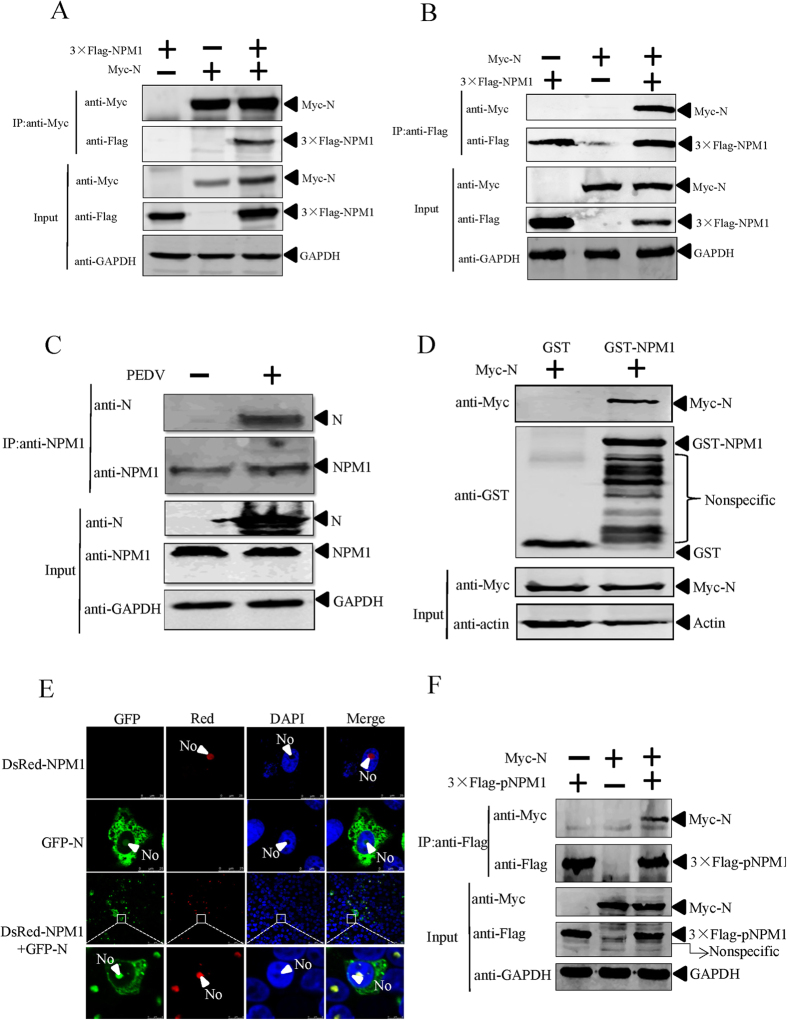
Interaction of PEDV N with NPM1. Co-IP of NPM1 with N protein. HEK293T cells were co-transfected with the indicated plasmids (+) or empty vectors (−) and the whole-cell lysates (WCL) obtained at 48 hpt were immunoprecipitated with anti-Myc (**A**) or anti-Flag (**B**) mAb. After separation by SDS-PAGE, proteins were detected by immunoblotting with the indicated antibodies. A 5% aliquot of WCL was also probed to confirm protein expression. The identities of the protein bands are indicated on the right. (**C**) Co-IP of PEDV N protein with endogenous NPM1. PEDV-infected (+) or mock-infected (−) Vero E6 cells were used for IP with anti-NPM1 protein mAb and immunoblotted with the indicated antibodies. The identities of the bands are shown on the right. (**D**) GST-pull down assay. Glutathione beads conjugated to GST or the GST-NPM1 fusion protein were incubated with recombinant Myc-N. After washing, proteins were eluted from the beads and SDS-PAGE was performed. The presence of N protein was detected by immunoblotting with anti-Myc mAb. GST and GST-NPM1 protein expression was confirmed by immunoblotting with mouse anti-GST mAb. (**E**) Co-localization of N protein with NPM1. Vero E6 cells were co-transfected with pAcGFP-N and pDsRed-NPM1. The PEDV N protein is colored green and the NPM1 fusion protein colored red. Merged images are also presented, and the position of the nucleus is indicated by DAPI (blue) staining in the merged images. The nucleolus (No) is arrowed where appropriate. Lower panels show boxed regions at high magnification. (**F**) Co-IP of HEK293T cells co-transfected with recombinant constructs encoding Myc-N and 3×Flag-tagged porcine NPM1.

The nucleolus is structurally divided into three major subcompartments: the fibrillar center, a dense fibrillar component, and a granular component^36^. Three distinct proteins that are mainly located in the nucleolus have been identified^37^,^38^: the major nucleolar protein fibrillarin, which is a component of a nucleolar small nuclear ribonucleoprotein that is involved in rRNA processing; NPM1, which is a putative ribosome assembly factor; and nucleolin, which is involved in the processing of precursor rRNA. To characterize the level of expression of N, NPM1, fibrillarin and nucleolin, encoding plasmids were constructed with Myc or 3×Flag at the N-terminus of each protein. Individual expression plasmids were transiently transfected into HEK293T cells, and their expression was assessed using western blotting (Fig. S1). As measured by western blotting using a monoclonal antibody (mAb) against N (see Supplementary Fig. S1A), Myc (see Supplementary Fig. S1B) or Flag (see Supplementary Fig. S1C) tag, individual expression constructs encoding N, NPM1 and fibrillarin proteins were expressed at different levels at the appropriate size, however, the construct encoding nucleolin failed to be expressed. To investigate the possible molecular target of N in the nucleolar, co-immunoprecipitation (Co-IP) experiments were performed. The result indicated that N protein showed an interaction with NPM1 in the Co-IP assay (Fig. 2A,B). To investigate whether N protein is able to interact with endogenous NPM1 in the context of PEDV infection, virus-infected Vero E6 cell lysates were immunoprecipitated with an anti-NPM1 mAb and probed for the presence of N protein with anti-N mAb. N protein was readily detected in PEDV-infected Vero E6 cells (Fig. 2C), indicating that N protein indeed interacts with endogenous NPM1 protein in PEDV-infected Vero E6 cells. However, the interaction of N protein with fibrillarin or nucleolin was not observed in the Co-IP assay (see supplementary Fig. S2A and B).

To verify and extend the binding data obtained in the Co-IP assay, we performed glutathione S-transferase (GST)-pull down experiments. The GST or GST-NPM1 protein was expressed in *Escherichia coli* and immobilized on glutathione-conjugated Sepharose beads. Beads carrying GST or GST-NPM1 were incubated with lysates from HEK293T cells transfected with pCMV-Myc-N. After thorough rinsing, the protein complex captured on the beads was solubilized, subjected to electrophoresis in a denaturing gel, and immunoblotted with anti-Myc or anti-GST antibody. As shown in Fig. 2D, the GST-NPM1 protein could pull down Myc-N. In contrast, GST alone did not pull down Myc-N. These data indicate that N protein can specifically interact with NPM1.

To examine the co-localization of N protein with NPM1, Vero E6 cells were co-transfected with plasmids expressing AcGFP-N and DsRed-NPM1 proteins, and the subcellular localization of N protein and NPM1 was examined by confocal microscopy (Fig. 2E). Imaging indicated that, as previously shown, the AcGFP-N protein localized to both the cytoplasm and the nucleolus, but not to the nucleus, in Vero E6 cells; DsRed-NPM1 protein localized to the nucleolus; and co-localization result showed 70.5 ± 14.2% of N protein positive cells were NPM1 positive in the nucleolus. Moreover, the interaction of N protein with porcine NPM1 protein also was validated in our studies (Fig. 2F). In total, these data indicated that N protein is able to interact with NPM1 protein.

### Amino acids 148–294 of N protein are responsible for binding to NPM1

**Figure 3:**
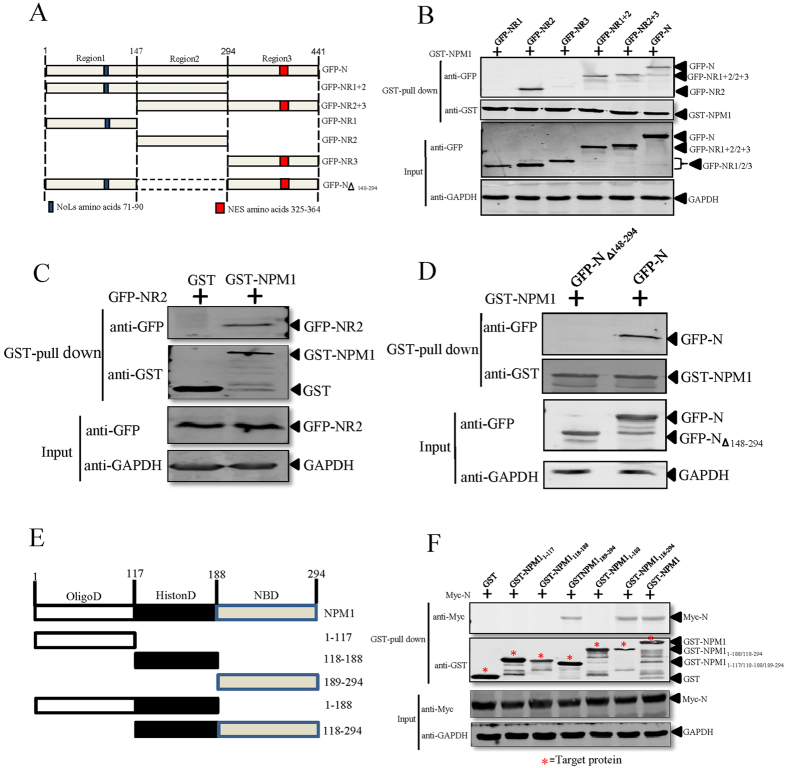
GST-pull down mapping of binding domains between N protein and NPM1. (**A,B,C** and **D**) GST-pull down mapping domains of N protein for NPM1 binding. (**A**) Schematic representation of full-length and deletion mutants of GFP-tagged N protein. (**B,C** and **D**) Glutathione beads conjugated to the GST-NPM1 fusion protein were incubated with full-length and deletion mutants of GFP-tagged N protein and the pulled down proteins were immunoblotted against GFP. (**E** and **F**) GST-pull down probing regions of NPM1 for N protein binding. (**E**) Schematic diagram of full-length and deletion mutants of GST-tagged NPM1. (**F**) GST-pull down assays were performed by incubation of purified GST-NPM1 or its deletion mutants with Myc-N protein. Pull down fractions were detected by immunoblotting against Myc mAb.

To define the specific region of N protein required for the interaction with NPM1, we used a series of GFP-tagged N protein deletions^15^ to map the NPM1 binding site on N protein (Fig. 3A). The GST-pull down assay revealed that GST-NPM1 bound to GFP-NR2, GFP-NR1+2, GFP-NR2+3 and GFP-N (Fig. 3B), but not to GFP-NR1 and GFP-NR3. In contrast, GST alone did not pull down GFP-NR2 (Fig. 3C). Furthermore, constructs lacking the NR2 domain (GFP-NΔ_148–294_) failed to interact with NPM1, suggesting that the NR2 domain of N protein is critical in binding to NPM1 (Fig. 3D).

### The C-terminal of NPM1 mediates its interaction with N protein

Various functional domains have been identified within NPM1, including an N-terminal oligomerization domain (OligoD) bearing chaperone activity, the C-terminal nucleic acid binding domain (NBD), and two central acid domains for histone binding (HistonD). To characterize further the interaction of NPM1 and N protein, we mapped the domains of NPM1 necessary for its association with N protein, based on the well-known functional domain of NPM1, and using a series of GST-tagged NPM1 deletion mutants (1–294, 1–117, 118–188, 189–294, 1–188, and 118–294) fused to GST (Fig. 3E). The results indicated that the C-terminal (aa 189–294) is essential for the association of NPM1 with N protein (Fig. 3F).

### NPM1 phosphorylation or sumoylation has no effect in mediating its binding to PEDV N protein

**Figure 4:**
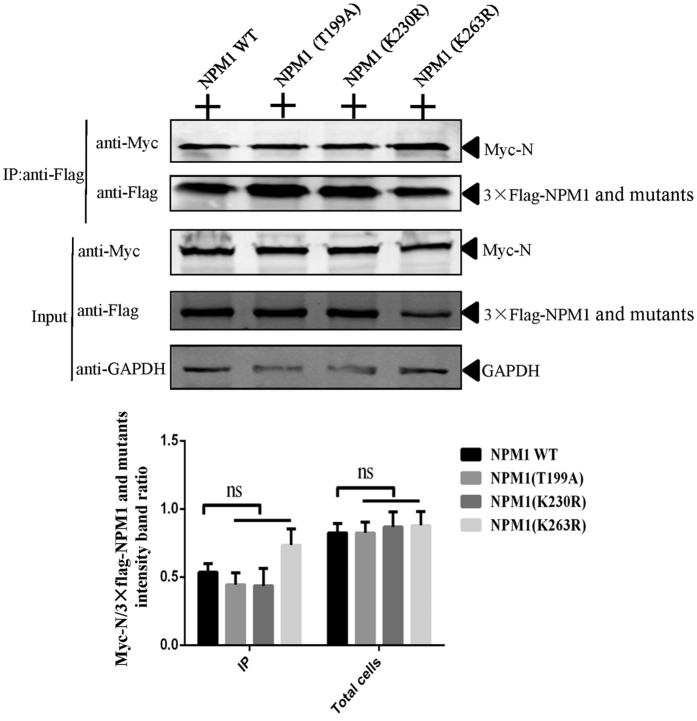
NPM1 phosphorylation or sumoylation has no effect in mediating its binding to PEDV N protein. HEK293T cells were co-transfected with the indicated plasmids, and the WCL obtained at 48 hpt were immunoprecipitated with anti-Flag mAb. After separation by SDS-PAGE, proteins were detected by immunoblotting with the indicated antibodies. A 5% aliquot of WCL was also probed to confirm protein expression. The identities of the protein bands are indicated on the right. Densitometric data for Myc-N/3×Flag-NPM1 and mutants from three independent experiments are expressed as mean ± SD.

It has been shown previously that CDK2/cyclin E-mediated phosphorylation of NPM1 on Thr-199 promotes dissociation of NPM1 from centrosomes, allowing the initiation of centrosome duplication^39^. Thus, when the T199A unphosphorylatable mutant is ectopically expressed, T199A binds continuously to centrosomes, resulting in suppression of centrosome duplication^40^. A recent study^41^ showed that NPM1 can be sumoylated on both Lys-230 and Lys-263 residues, although Lys 263 is the major sumoylation site. Mutation of K263 alters its subcellular distribution, and K263R mutation makes NPM1 susceptible to caspase-3 cleavage and decreases cell proliferation. Intriguingly, Thr-199, Lys-230 and Lys-263 are all located in the C-terminal domain of the NPM1 protein, which is essential for the interaction of NPM1 with N protein. To explore whether Thr-199, Lys-230 and Lys-263 have a role in the association between NPM1 and N protein, we co-transfected various 3×Flag-NPM1 constructs into HEK293T cells with Myc-N. Co-IP assays demonstrated that the unphosphorylated T199A, unsumoylated K230R and K263R were able to bind Myc-N, and had either moderate effects or no effect on N protein binding (Fig. 4).

### N protein is imported into the nucleolus independently of NPM1

**Figure 5:**
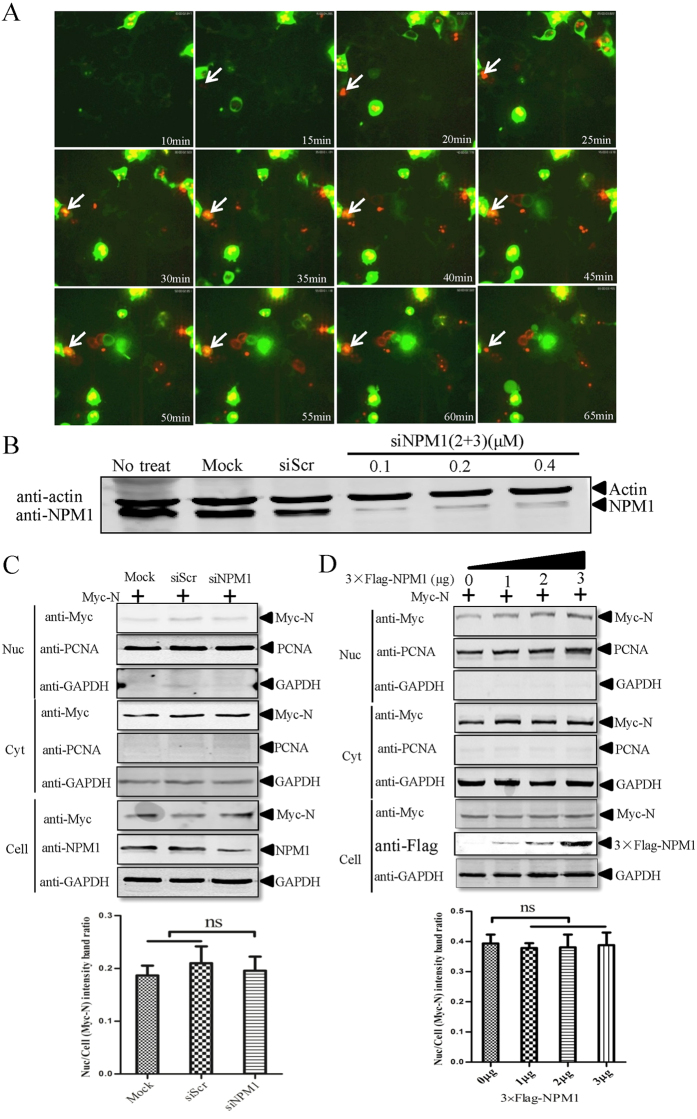
N protein is imported into the nucleolus independently of NPM1. (**A**) The NPM1 fusion protein as a marker of the nucleolus is colored red. The nucleolar localization of N in transfected cells was clearly observed at 42–42.5 hpt. As shown, a small amount of N was observed in the nucleolus at 42 hpt (t = 30 min). N protein accumulated continuously in the nucleolus of transfected cells until t = 60 min and was exported from the nucleolus at t = 61–65 min. This figure shows snapshots of the cells from the time-lapse movie (Video S3 in the supplemental materials). Data are representative of one of three independent experiments. Real-time visualization of the kinetics of the nucleolar localization of N protein indicated that the process was rapid, taking only 30 min in total. (**B**) Knockdown of NPM1 protein levels following siRNA treatment. Vero E6 cells transfected with no siRNA (Mock), scrambled siRNA (siScr), left untreated (No treat) or with different concentrations (mM) of siRNAs targeting NPM1 (siNPM1) (as indicated at the top of each lane) were harvested 48 hpt. Endogenous NPM1 protein levels were detected by immunoblotting using antibodies directed against the indicated proteins. (**C** and **D**) Western blot analysis of Myc-N protein in nuclear and cytoplasmic fractions of NPM1-knockdown cells (**C**) or Ectopic NPM1-overexpression cells (**D**) at 48 hpt with anti-GAPDH mAb, anti-PCNA mAb, anti-NPM1 mAb, anti-Myc mAb and anti-Flag mAb. Nuc, nuclear fraction; Cyt, cytoplasmic fraction; cell, whole cells. Densitometric data for Nuc/Cell (Myc-N) from three independent experiments are expressed as mean ± SD.

NPM1 localizes in granular regions of the nucleolus^42^, is associated with preribosomal particles^43^, and forms pentamers that may be important for the assembly of ribosomes^44^. NPM1 has the ability to shuttle between the nucleus and the cytoplasm^45^; it binds to nuclear/nucleolar localization signal containing peptides^45^, and thus serves as a shuttle protein in nuclear/nucleolar import. Interestingly, in this study we also found that the NPM1 interacts with N protein, suggesting that NPM1 may serve as a shuttle protein for transport of N protein into the nucleolus, although the interaction domain of NPM1–N (aa 148–294) is not within the fragment of N protein that contains the nucleolus localization signal (aa 70–90). It is hypothesized that transport of N protein into the nucleolus depends on the movement of NPM1 between the nucleus and the cytoplasm. To examine this hypothesis, we co-transfected HEK293T cells with pAcGFP-N and pDsRed-NPM1, and time-lapse images were acquired at 12–72 hpt at 5 min intervals. The transport into the nucleolus, interaction with NPM1 and export of N protein in transfected cells were clearly observed at 42–43 hpt. As shown in Fig. 5A, NPM1 protein appeared first in the nucleolus (t = 10 min) and then a small amount of N was observed in the nucleolus at 42 hpt (t = 30 min). N protein accumulated continuously in the nucleolus of transfected cells and interacted with NPM1 until t = 30–55 min, and was exported from the nucleolus at t = 55–60 min. Real-time visualization of the kinetics of nucleolar import, interaction with NPM1 and export of N protein indicated that the process was rapid, taking only 30 min in total, thereby ruling out the possibility that the nucleolar localization of N protein is NPM1 independent. The time-lapse video showing the kinetics of nucleolar translocation of N protein and its interaction with NPM1 is provided as supplementary material (Video S3 in the Supplementary materials). To corroborate our findings, we first tested whether depletion of NPM1 by specific siRNAs resulted in reduced nuclear import of N. For this purpose, three pairs of NPM1 siRNAs were synthesized. These siRNAs were transfected into Vero E6 cells, and it was found that NPM1 siRNA (2+3) reduced the level of expression of NPM1 (Fig. 5B). In addition, we examined the viability of cells receiving siRNA using the CCK-8 assay. The results showed that there was no difference between NPM1 RNAi and RNAi control in terms of the viability of transfected cells (see Supplementary Fig. S4). As shown in Fig. 5C, transport of N protein was hardly affected in cells with reduced NPM1 levels in comparison with that in the cells treated with a scramble siRNA (siScr), mock-treated cells. As a control, we also co-transfected Vero E6 cells with Myc-N and 3×Flag-NPM1, but did not observe a stimulation of nuclear import of Myc-N with the increased expression of the NPM1 protein (Fig. 5D).

### Overexpression of NPM1 enhances PEDV growth, and PEDV infection increases NPM1 expression

**Figure 6:**
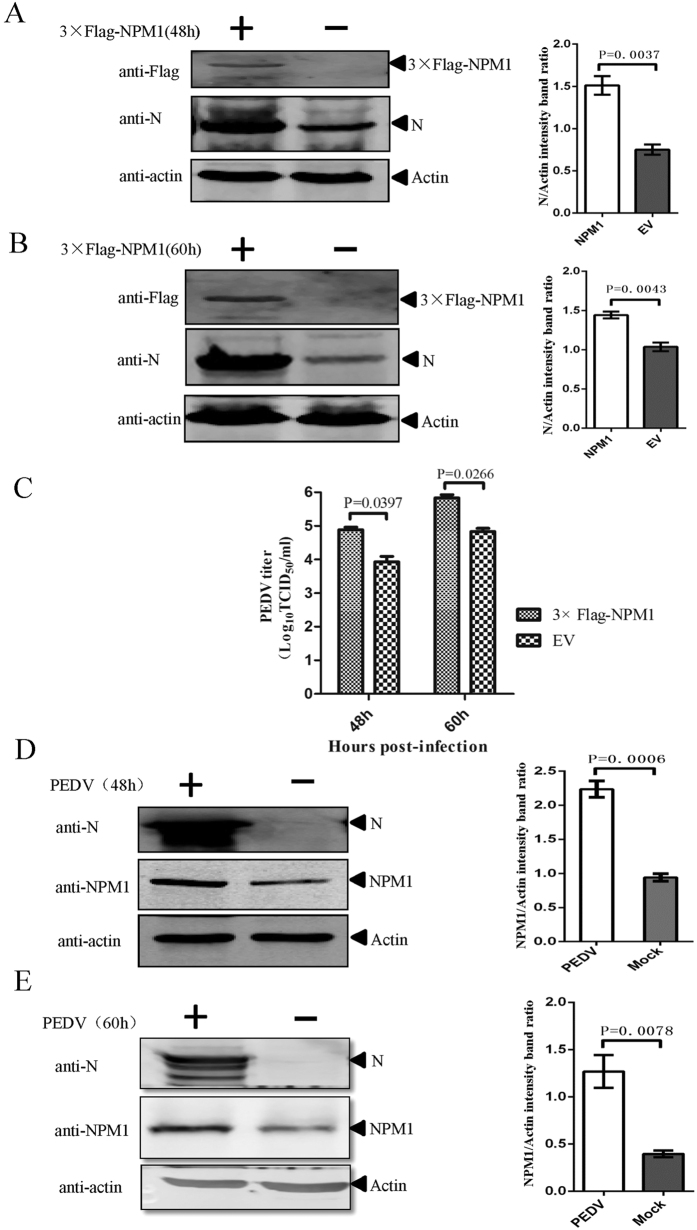
Overexpression of NPM1 promotes N protein expression and PEDV growth. (**A** and **B**) Ectopic expression of NPM1 in Vero E6 cells. Vero E6 cells were transfected with p3×Flag-NPM1 (+) or empty vector (–) for 12 h, followed by infection with PEDV at a MOI of 0.1 for 48 h (**A**) or 60 h (**B**). The levels of expression of 3×Flag-NPM1, N, or β-actin (loading control) (**A** and **B**) proteins in cell lysates were analyzed by western blot of cell lysates at the indicated time points after infection. Densitometric data for N/actin from three independent experiments are expressed as mean ± SD. (**C**) Promotion of PEDV growth in NPM1-overexpressing cells. Transfection and infection conditions were as described for panel A and B, and the titers of virus in the supernatants collected at 48 and 60 hpi were determined by the Reed–Muench method. Error bars represent the standard errors of the means from three independent experiments. P values are indicated on the bars. (**D** and **E**) Expression of endogenous NPM1 protein in Vero E6 cells infected with PEDV at various time points. Cells were infected with PEDV at MOI of 0.1 for 48 h or 60 h. The levels of expression of N, NPM1, or β-actin (loading control) (**D** and **E**) proteins in cell lysates were analyzed by western blot of cell lysates at the indicated time points after infection. Densitometric data for NPM1/actin from three independent experiments are expressed as mean ± SD.

The observation that NPM1 interacts with PEDV N protein prompted investigation of the relevance of this interaction to the PEDV life cycle. Vero E6 cells were transfected transiently with 3×Flag-NPM1 and subsequently infected with PEDV. In these cells, 3×Flag-NPM1 could be readily detected, and the expression of N protein was increased (Fig. 6A and B). The results showed that overexpression of NPM1 results in upregulation of N protein expression when compared with the empty vector. An increase in viral titer was also observed in the supernatants of these cells (Fig. 6C). Furthermore, the expression of NPM1 in Vero E6 cells infected with PEDV was assessed, and the results indicated that PEDV infection increases the expression of endogenous NPM1 (Fig. 6D and E).

### Knockdown of NPM1 in Vero E6 cells results in inhibition of PEDV growth

**Figure 7:**
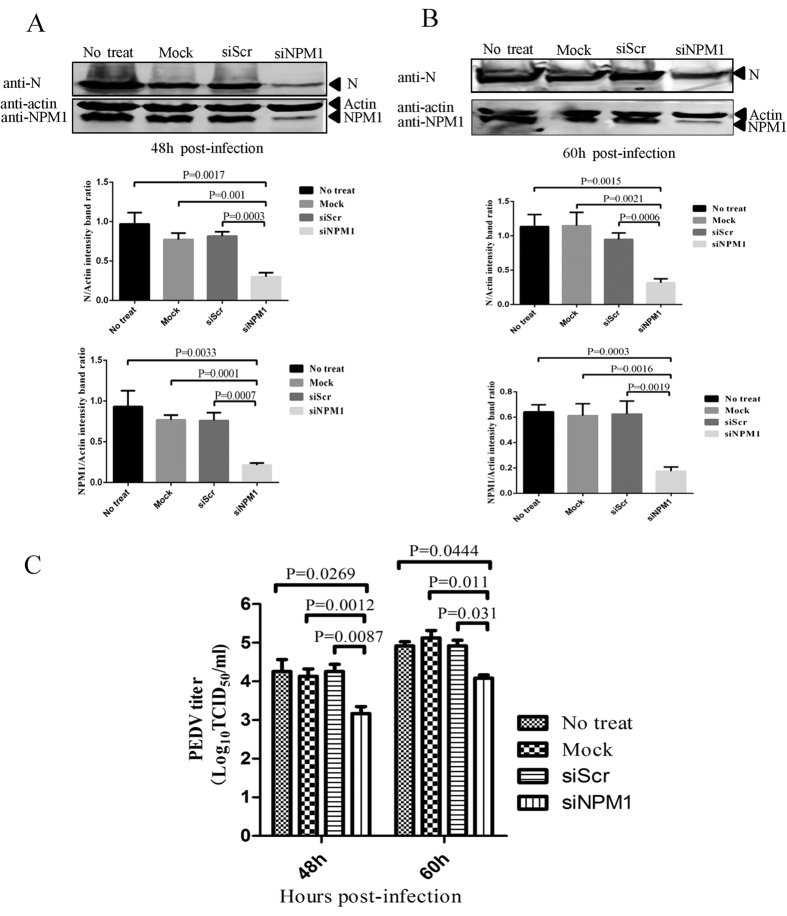
Knockdown of NPM1 inhibits PEDV growth and N protein expression. (**A** and **B**) PEDV N protein is reduced in NPM1-knockdown cells. Vero E6 cells treated with 200 nM siNPM1, No treat, Mock or siScr for 48 h were infected with PEDV for 48 h (**A**) or 60 h (**B**). Endogenous NPM1 was detected by western blot with anti-NPM1 mAb and PEDV N protein was detected by western blot with anti-N protein mAb. Densitometric data for N/actin and NPM1/actin from three independent experiments are expressed as mean ± SD. (**C**) PEDV titers in NPM1-knockdown cells. siRNA transfection and virus infection were performed as in panel A and B, and the virus titers in supernatants collected at 48 and 60 hpi were determined by the Reed–Muench method. Error bars represent the standard error of the mean from three independent experiments. P values are indicated above the bars.

Given that overexpression of NPM1 significantly affected PEDV replication in Vero E6 cells, it was interesting to investigate whether knockdown of NPM1 also affected PEDV replication. To this end, siRNA-mediated knockdown of NPM1 in Vero E6 cells infected with PEDV was investigated. As shown in Fig. 7A and B, knockdown of NPM1 resulted in downregulation of N protein expression at 48 or 60 hpi, accompanied by a significant reduction of viral load in the cell culture supernatants in comparison with that in the cells treated with a scramble siRNA (siScr), mock-treated cells, or normal Vero E6 cells (no treatment) (Fig. 7C). This indicated that growth of PEDV was arrested in cells with a reduced level of NPM1. Together with the results of the overexpression experiments, these data highlight the synergistic action of cellular NPM1 expression on PEDV replication and N protein expression.

### N protein binding prevents proteolytic cleavage of NPM1 and enhances cell survival

**Figure 8:**
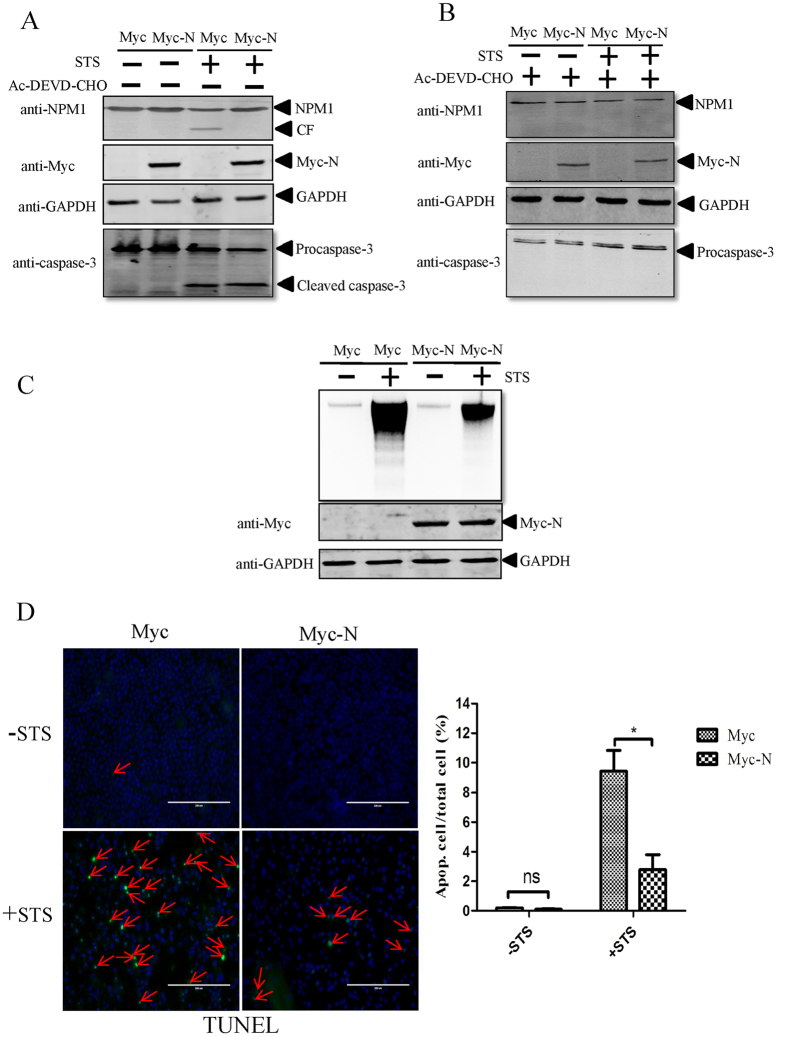
N protein binding prevents NPM1 proteolytic cleavage and enhances cell survival. (**A** and **B**) N protein binding prevents NPM1 proteolytic cleavage. Vero E6 cells were transfected with pMyc-N or empty vector for 24 h and then treated with or without 100 μM of Ac-DEVD-CHO (caspase-3 inhibitor) for 6 h. The cells were treated with or without 250 nm of STS for 18 h. The western blots were probed for freshly extracted proteins with antibodies against NPM1, Myc, GAPDH and caspase-3. Verification of Myc-N induction and equal sample loading are shown by anti-Myc and anti-GAPDH mAbs. CF, cleavage fragment. (**C**) N protein enhances the antiapoptotic effect of NPM1. Vero E6 cells were transfected with pMyc-N or empty vector for 30 h and then treated with or without 250 nm of STS for 18 h. Genomic DNA was loaded on to a 2% agarose gel. Verification of Myc-N induction and equal sample loading are shown by anti-Myc and anti-GAPDH mAbs. (**D**) Vero E6 cells were transfected with pMyc-N or empty vector for 30 h and then treated with or without 250 nm of STS for 18 h, then TUNEL and DAPI staining to examine the apoptotic cell death. Statistical results represent means ± SD of apoptotic cell counts from six different fields (right).

Programmed cell death, or apoptosis, is an essential event in animal development and is observed in many developing tissues in both invertebrates and vertebrates. The activation of the caspase family is a central event in apoptosis. Downstream caspases include caspase-3, the precursor form of which is predominantly synthesized in the cytosol^46^. Activated caspase-3 can be translocated from the cytoplasm into the nucleus^47^. Caspase-3 is activated by upstream caspases and then cleaves many intracellular target proteins to induce apoptotic cell death; for example, NPM1 is a substrate of caspase-3^48^. To explore whether N protein binding has any role in mediating the apoptotic cleavage of NPM1, we transiently transfected Myc-N and empty vector into Vero E6 cells and then treated with or without 100 μM of Ac-DEVD-CHO (caspase-3 inhibitor). The cells induced apoptotic cleavage of NPM1 by treatment with an apoptosis inducer, staurosporine (STS), or not. Apoptotic stimulus clearly demonstrated that NPM1 protein cleavage and occurred in empty vector cells following STS treatment. In contrast, NPM1 was almost intact when N was overexpressed in Vero E6 cells with or no STS treatment, highlighting that nucleolus-targeted N prevents apoptotic degradation of NPM1 (Fig. 8A). Meanwhile, cleaved caspase-3 was found in STS treated cells (Fig. 8A). Furthermore, Ac-DEVD-CHO, a caspase-3 inhibitor, was able to restore the STS-induced NPM1 and caspase-3 protein cleavage (Fig. 8B). This suggests that interaction with N protein protects NPM1 from apoptotic degradation. A number of studies have indicated that NPM1, one of the major nucleolar phosphoproteins, is involved in the regulation of nucleolar function during cellular differentiation^49^ and in antiapoptosis^48^. To evaluate whether the N–NPM1 complex is important in prevention of apoptosis, we studied Vero E6 cells transiently expressing Myc or Myc-N and induced apoptosis by STS. Compared with cells with no STS treatment, a DNA fragmentation assay revealed that overexpression of PEDV N protein slightly diminished DNA degradation. By contrast, robust DNA fragmentation was detected in cells transiently expressing Myc tag (Fig. 8C). To evaluate further the antiapoptotic effect of N protein *in vivo*, we examined the sensitivity of Vero E6 cells transfected with Myc-N or empty vector and with induction of apoptosis by treatment with STS or not. DAPI and TUNEL staining of the nucleus revealed that Myc-N transfected cells displayed greater viability after stimulation by STS and were markedly less sensitive to STS-induced apoptosis than empty vector transfected cells (Fig. 8D). Collectively, these data demonstrate that the N–NPM1 interaction plays an essential role in protecting cells from apoptotic degradation, thus promoting cell survival.

## Discussion

The interaction of viral proteins with nucleolar antigens may explain why viral proteins have been observed in the nucleolus and may also explain the viral exploitation of nucleolar function, leading to alterations in host cell transcription and translation, and disruption of the host cell cycle to facilitate viral replication. Our previous^15^ and current studies (Fig. 1) indicate that the PEDV N protein is actively transported to the nucleolus during the time course of PEDV infection. The function of N protein during PEDV infection is thought to require interaction with cellular proteins, therefore in this study we investigated whether the PEDV N protein interacts with three major nucleolar antigens: NPM1, fibrillarin and nucleolin. Interaction with one or all of these antigens may explain our previous observations that PEDV N protein is localized to the nucleolus^15^. Proteins that localize to the nucleolus have been reported to be involved in cell growth, the cell cycle and cell survival^25^,^41^. In the current studies, we also wished to investigate whether interaction of the N protein with nucleolar proteins affects PEDV replication.

This study was based on different lines of evidence, reflecting both *in vivo* and *in vitro* situations, and demonstrated that PEDV N protein is able to associate with the major nucleolar protein NPM1 of Vero E6 cells (Fig. 2); we failed to detect an interaction with the fibrillarin or nucleolin (See Supplementary Fig. S2). The N protein also interacted with porcine NPM1 (Fig. 2F). In the immunoprecipitation (Fig. 2A,B) and the GST-NPM1 fusion protein pull down assay (Fig. 2D), both *in vitro* translated and cellular NPM1 were shown to interact with N protein. The immunoprecipitation experiment in PEDV-infected Vero E6 cells provided further support for the *in vivo* binding of NPM1 and N protein (Fig. 2C). The confocal microscopy analysis showed the co-localization of NPM1 and N in the nucleolus (Fig. 2E), and the *in vitro* binding studies utilizing deletion mutants of N or NPM1 defined the binding sites of these two proteins. As shown in Fig. 3B,3C and 3D, apart from the full-length protein, only the N variants containing the NR2 fragment, NR2, NR1+2 and NR2+3, but not the C-terminal or N-terminal fragment, were able to bind to NPM1, suggesting that the domain that interacts with NPM1 is within amino acid residues 148–294 of N. Interestingly, this region of N consists of an SR-domain, containing serine and arginine residues^50^, and is involved in cell signaling and post-translational modifications such as phosphorylation^51^. NPM1 has been reported to bind to the arginine-rich basic region of the human T-cell leukemia virus protein Rex^52^, and to the HIV proteins Rev^23^ and Tat^53^. However, further studies will be required to determine more precisely the location of the interaction domain and the specific amino acid residues that participate in the interaction.

The protein NPM1 is multifunctional and exhibits nucleic acid binding, ribonuclease activity, and molecular chaperone activity^54^,^55^. These three activities reside in nearly independent but partially overlapping segments of the polypeptide chain^56^. The N-terminal nonpolar region and the acidic region of the middle portion of NPM1 are important for its chaperone activity, and the C-terminal is essential for nucleic acid binding^56^. Interestingly, analysis of the binding sites of targeting proteins on NPM1 has revealed that most of them reside in the C-terminal portion of the molecule. For example, NPM1 binds to the nucleolar proteins P120^57^, nucleolin^58^, and Tat^53^ through a fragment of NPM1 containing amino acids 187–215 or 194–239. This is in accordance with the interaction of N protein with NPM1, which occurs at the C-terminal portion of NPM1 (Fig. 3F). The binding region of NPM1 for viral proteins HDAg^59^ and Rex^52^ has been localized to these acidic regions. Therefore, these results suggest that the interaction of NPM1 with N protein is similar to the interactions with nucleolar protein p120, nucleolin, and Tat, but is different from the interaction of NPM1 with the viral proteins HDAg and Rex.

The localization of viral proteins to the nucleolus generally occurs through the interactions of basic regions on the viral protein with stretches of acidic residues on nucleolar proteins such as NPM1 and nucleolin^60^,^61^. Upon binding to viral protein in the cytoplasm or in the nucleus, NPM1 and nucleolin function as shuttle proteins, directing the transport of viral proteins across the nuclear pore complex into the nucleoplasm and then to the nucleolus. However, the transport of N protein from the cytoplasm into the nucleolus was not dependent on the shuttle protein NPM1 in this study (Fig. 5 and Video S3 in the Supplementary materials). One possible reason is that the N protein may bind to importin α and importin β; both play essential roles in the nuclear transport of proteins through the nuclear pore complex. Whether other viral proteins or host factors are involved in the nuclear transport of PEDV N protein needs to be determined in the future. The role of N protein during its interaction with NPM1 may represent a unique function of N protein in the nucleolus.

NPM1 is a multifunctional protein involved in many cellular and viral activities. In particular, NPM1 interacts with viral proteins from several different viruses and promotes viral replication cycles. Interaction between NPM1 and adenoviral protein V promotes virus assembly during virion maturation^21^. NPM1 also forms a complex with hepatitis delta virus (HDV) antigens to enhance replication of HDV RNA^59^. In this study, we also demonstrated the synergistic action of cellular NPM1 expression on PEDV replication and N protein expression (Figs 6 and 7). These interactions link NPM1 with the viral life cycle as an important protein for viral replication.

Apoptosis is an important mechanism by which virus-infected cells are eliminated from the host. Accordingly, many viruses have evolved strategies to prevent or delay apoptosis in order to provide a window of opportunity in which virus replication, assembly and egress can take place. Interfering with apoptosis may also be important for establishment and/or maintenance of persistent infections. With few exceptions, most studies of virus-encoded antiapoptotic proteins have focused on DNA viruses. The known exceptions are the picornavirus-encoded proteins leader and 2BC^62^,^63^, as well as the rubella virus capsid protein^64^. Although infection with these viruses induces apoptosis in many cell lines, this is generally observed late in the infection process. In this report, we have demonstrated that the major isoform of the PEDV N protein in infected cells functions to block apoptosis. The protective capacity of N protein is dependent on interaction with NPM1; it protects it from proteolytic cleavage, enhancing cell survival, and positively regulates PEDV replication and growth.

In summary, the key findings of this study are the identification of nucleolus protein NPM1 as a novel interacting partner of the PEDV N protein. That NPM1 promotes PEDV growth is due to N protein inhibition of caspase-3-mediated cleavage of NPM1, which prevents proteolytic cleavage of NPM1 and enhances host cell survival. The identification and characterization of the interaction of PEDV N protein with NPM1 with the resultant alteration in host cell survival may facilitate the development of vaccines and therapeutics for use in pigs.

## Materials and Methods

### Cells, viruses, and virus titer assays

Human Embryonic Kidney 293 T (HEK293T) cells and Vero E6 cells were purchased from ATCC, grown in Dulbeccos modified Eagle’s medium (DMEM) supplemented with 10% heat-inactivated fetal bovine serum (FBS) and penicillin–streptomycin, and incubated at 37 °C in 5% CO_2_. The PEDV strain CV777 was propagated in PEDV-infected Vero E6 cells. Virus titers in the culture supernatants of PEDV-infected Vero E6 cells were determined by the Reed–Muench method.

### Plasmids

The plasmids expressing GFP-tagged N, NR1, NR2, NR3, NR1+2 and NR2+3 have been described previously^15^. The NPM1 and fibrillarin genes were amplified from pDsRed-NPM1^15^ and the genome of Vero E6 cells, respectively. Both were cloned into a p3×Flag-CMV-10 vector (E7658; Sigma) with the *Eco*RI and *Kpn*I restriction enzymes. The PEDV N protein gene was cloned into the pCMV-Myc vector (631604; Clontech) with the *Sal*I and *Kpn*I restriction enzymes to generate the pMyc-N plasmid. For bacterial expression of the GST-tagged NPM1 protein, the NPM1 protein gene region was subcloned into the pGEX-6p-1 vector (28–9546–48; GE Healthcare), creating pGEX-NPM1. A series of mutant forms of NPM1 was generated from pDsRed-NPM1 by conventional PCR with the mutagenesis primers listed in Table 1. Additionally, ΔNR2, NPM1 (T199A), NPM1 (K230R) and NPM1 (K263R) were generated by overlapping PCR. All plasmids were verified by sequencing.

### Plasmid DNA transfection

Cells in six-well plates (Corning) cultured at 37 °C in a humidified incubator with 5% CO_2_ were transfected with the respective plasmids (3 μg each) using the Attractene Transfection Reagent (301005; QIAGEN) according to the manufacturer’s instructions. At 6 h post-transfection (hpt), the transfection mixture was replaced with complete growth medium and incubated for an additional 48 h before being used for assays.

### Virus infection and treatment

After DNA or small interfering RNA (siRNA) transfection, cells were infected with PEDV strain CV777 at a multiplicity of infection (MOI) of 0.1. After 1 h, the viral inoculum was removed and the infected cells were washed three times with phosphate-buffered saline (PBS; pH7.4) and re-fed with DMEM containing 1 μg/ml trypsin. At various time points post-infection, cell-free culture supernatants and cell lysates were harvested and stored at −80 °C until use.

### Preparation of nuclear and cytoplasmic fractions

Nuclear and cytoplasmic fractions were prepared as described previously^65^. Briefly, treated Vero E6 cells were scraped into ice-cold PBS, centrifuged at 3000 × *g*, and resuspended in ice-cold buffer A (10 mM HEPES [pH 7.9], 10 mM KCl, 0.1 mM EDTA, 0.1 mM EGTA, 1 mM dithiothreitol), and then Nonidet P-40 (final concentration, 0.1%) was added. The cells were lysed by five strokes of a Dounce tissue homogenizer (Bellco Glass). The nuclear fraction was pelleted by centrifugation at 12,000 × *g* for 30 s at 4 °C. The supernatant was used as the cytoplasmic fraction. To ensure that the subcellular fractions were separated properly, subcellular lysates were verified by the antibodies against the corresponding fractions. These antibodies were anti-glyceraldehyde-3-phosphate dehydrogenase (GAPDH) for the cytoplasm and anti-proliferating cell nuclear antigen (PCNA) for the nucleus.

### GST-pull down assays

For the GST-pull down assays, GST or GST-NPM1 protein produced in *Escherichia coli* BL21 (DE3) cells was conjugated to glutathione beads (10049253; GE Biosciences) and blocked for 1 h in 5% bovine serum albumin. The beads were then washed three times with TIF buffer (20 mM Tris-HCl [pH 8.0], 150 mM NaCl, 1 mM MgCl_2_, 0.1% Nonidet P-40, 10% glycerol, 0.1 mM dithiothreitol, 1 mg/ml protease inhibitor) and incubated for 6 h at 4 °C with recombinant Myc-tagged N harvested from transfected HEK293T cells. The beads were washed at least five times with TIF buffer, followed by elution and detection of the proteins by sodium dodecyl sulfate–polyacrylamide gel electrophoresis (SDS-PAGE) and immunoblotting.

### Co-immunoprecipitation (Co-IP)

HEK293T cells were transfected with the indicated constructs as described above. The transfected cells were harvested at 48 hpt, washed three times with cold PBS (pH 7.4), and lysed with IP lysis buffer (87788; Thermo) containing 1 mM phenylmethylsulfonyl fluoride (PMSF) and 1 mg/ml protease inhibitor cocktail (04693132001;Roche) at 4 °C for 30 min. Clarified extracts were precleared with protein A/G beads (SC-2003; Santa Cruz) and then incubated with protein A/G beads plus anti-Flag (F3165; Sigma), Myc (C3956; Sigma) or NPM1 (PLA0253; Sigma) mAb for 6–8 h. The beads were then washed with IP lysis buffer and boiled in sample buffer, and the proteins were subjected to SDS-PAGE, followed by immunoblotting analysis with anti-Flag, anti-Myc and anti-N mAb.

### Real-time visualization of N nucleolar transport

To visualize the nucleolar transport of N, HEK293T cells were seeded in glass-bottomed 35-mm dishes (MatTek) and then co-transfected with pAcGFP-N and pDsRed-NPM1. Live-cell fluorescence images of transfected HEK293T cells were recorded (every 5 min) between 12 and 72 hpt using a TCS SP5 confocal microscope (Leica Laser Technik; Germany) equipped with a 60× oil objective. All images were acquired with 500-ms exposures under the same illumination conditions and analyzed using LAS AF 1.8.2 software (Leica Laser Technik; Germany).

### RNA interference

SiRNAs targeting NPM1 were used at a final concentration of 200 nM, unless otherwise stated. Cells were transfected with siRNAs with X-tremeGENE siRNA transfection reagent (4476093001; Roche) as described previously^66^ The siRNA target sequences of NPM1 were GGAAGATGCAGAGTCAGAATT (siNPM1–2) and GGAAGCCAAGTTCATCAATTT (siNPM1–3). Western blotting was used to analyze endogenous NPM1 protein production with anti-NPM1 mAb.

### Confocal imaging

Vero E6 cells were seeded on microscope slide coverslips, which were set in 35-mm diameter dishes, and grown to a confluence of ~50%. At 48 hpt, the cells were fixed with 4% paraformaldehyde for 30 min. Then the nucleus was stained with 4′,6-diamidino-2-phenylindole (DAPI) (0.05 μg/mL) (D9542; Sigma) for 15 min and analyzed by laser confocal scanning microscopy (Leica Laser Technik; Germany).

### Western blotting analysis

Total cellular proteins were extracted with the RIPA lysis buffer (R0278; Sigma) and the concentrations were determined with a Pierce^®^ BCA Protein Assay Kit (23225; Thermo). The total proteins (100 μg) were subjected to SDS-PAGE, and separated protein bands were electro-transferred onto a nitrocellulose membrane (66485; Pall) using a semidry blotter (Bio-Rad). The membrane was soaked in blocking buffer (PBS containing 5% nonfat milk) for 2 h and then reacted with the indicated antibodies: mouse anti-GAPDH mAb (1:10,000) (G8795; Sigma), mouse anti-PCNA mAb (1:200) (BM0104; BOSTER), mouse anti-N mAb (1:500), mouse anti-GST mAb (1:1000) (AG768; Beyotime), mouse anti-actin mAb (1:5000) (A5441; Sigma), mouse anti-GFP mAb (1:10,000) (66002–1–1 g; Proteintech), rabbit anti-Flag mAb (1:2000) (20543–1-AP; Proteintech), or mouse anti Myc mAb (1:2000) (66004–1–1 g; Proteintech), rabbit anti-caspase-3 mAb (1:1000) (AC030; Beyotime). The proteins were revealed by IRDye 800CW goat anti-mouse lgG (H+L) (1:10,000) (926–32210; LiCor BioSciences) and IRDye 680RD goat anti-rabbit lgG (H+L) (1:10,000) (926–68071; LiCor BioSciences), and thereafter the blots were visualized using an Odyssey infrared imaging system (LiCor BioSciences). Quantification of band intensities by densitometry was carried out using the Image J software.

### DNA fragmentation assay

Oligonucleosomal fragmentation of genomic DNA was investigated as described below. Briefly, 3 × 10^6^ cells in 10 ml of medium were incubated with 250 nM of staurosporine (STS) for 18 h. After incubation, the cells were lysed on ice for 60 min in 500 μl lysis buffer (0.02% SDS/1% Nonidet P-40/0.2 mg/ml proteinase K in PBS). Genomic DNA was extracted by the phenol/chloroform method. The pellet was dissolved in 50 μl of TE buffer (10 mg/ml RNase) for 2 h at 37 °C. A total of 10 μg of DNA was loaded on a 2% agarose gel and visualized under UV light.

### Terminal deoxynucleotidyl transferase-mediated dUTP-biotin nick end labelling (TUNEL) assay

The TUNEL assay was performed using the *In Situ* Cell Death Detection Kit, Fluorescein (11684795910;Roche), following the manufacturer’s instructions. In brief, Vero E6 cells treated or untreated with STS were fixed in 4% paraformaldehyde for 20 min at room temperature, washed with PBS, and permeabilized with freshly prepared 0.1% Triton X-100 and 0.1% sodium citrate for 2 min on ice. After washing with PBS, the cells were overlaid with 100 μl of TUNEL reaction mixture, according to the manufacturer’s instruction, and incubated for 1 h at 37 °C. Finally, the cells were washed with PBS, then the nucleus was stained with DAPI for 15 min and directly analyzed under a fluorescence microscope using an exciting wavelength in the range of 450–500 nm (488 was used in this experiment) and detection in the range of 515–565 nm. Quantification of the green fluorescence-positive cells was performed by taking the average of at least six fields of view.

### Cell viability assay

The cell viability assay was performed using the cell counting kit-8 (CCK-8) (CK04; Dojindo) according to the manufacture’s protocol. In brief, Vero E6 cells were seeded in a 96-well plate at a density of 10,000 per well and incubated at 37 °C for 24 h. The cells were transfected with siRNAs (or not), and the plates were incubated for 48 h. Subsequently, 10 μl of CCK-8 was added to each well, and the cells were further incubated for 2 h. The optical density at 450 nm was measured. The viability of the treated cells was expressed as a percentage relative to the untreated cells.

### Statistical analysis

Statistical analysis was performed using SPSS 19.0 software. Variables are expressed as mean ± SD. Student’s t test and one-way analysis of variance (ANOVA) were used to compare viral titers. A P value of < 0.05 was considered significant.

## Additional Information

**How to cite this article**: Shi, D. *et al*. Nucleocapsid Interacts with NPM1 and Protects it from Proteolytic Cleavage, Enhancing Cell Survival, and is Involved in PEDV Growth. *Sci. Rep.* **7**, 39700; doi: 10.1038/srep39700 (2017).

**Publisher's note:** Springer Nature remains neutral with regard to jurisdictional claims in published maps and institutional affiliations.

## Supplementary Material

Supplementary Information

Supplementary Video S3

## Figures and Tables

**Table 1:** Primers used in this study.

	Primers	Sequences (5′-3′)	Vectors
N	Myc-U	GCGTCGACCATGGCTTCTGTCAGCTTT (*Sal*I)	pCMV-Myc
	Myc-L	GCGGTACCTTAATTTCCTGTGTC (*Kpn*I)	
ΔNR2	GFP-U	GCACTCGAGCTATGGCTTCTGTCAGCTTTC (*Xho*I)	pAcGFP-C1
	GFP-L	GCCGGTACCTTAATTTCCTGTATCGAAGAT (*Kpn*I)	
	ΔNR2-2U	CTCCTGCTTCACGTACAAATTCGTTCGGACCCAGGGGGGGCTTCA	
	ΔNR2-2L	AGCCCCCCCTGGGTCCGAACGAATTTGTACGTGAAGC	
NPM1/pNPM1	3×Flag-NPM1/pNPM1-U	GCGAATTCAATGGAAGATTCGATGG (*Eco*RI)	p3×Flag-CMV-10
F23	3×Flag-NPM1pNPM1-L	GCGGTACCTTAAAGAGACTTCCTCCA (*Kpn*I)	p3×Flag-CMV-10
	3×Flag-F23-U	GCGAATTCAATGAAGCCAGGATTCAG (*Eco*RI)	
	3×Flag-F23-L	GCGGTACCTCAGTTCTTCACCTT (*Kpn*I)	
NPM1	GST-NPM1-U	GCGAATTCATGGAAGATTCGATGGAC (*Eco*RI)	pGEX-6P-1
	GST-NPM1-L	GCCTCGAGTTAAAGAGACTTCCTCCA (*Xho*I)	
NPM1_1–117_	GST-NPM1_1–117_-U	GCGAATTCATGGAAGATTCGATGGAC (*Eco*RI)	pGEX-6P-1
	GST-NPM1_1–117_-L	GCCTCGAGTTATACTAAGTGCTGTCCA (*Xho*I)	
NPM1_118–188_	GST-NPM1_118–188_-U	GCGAATTCGCTGTGGAGGAAGATG (*Eco*RI)	pGEX-6P-1
	GST-NPM1_118–188_-L	GCCTCGAGTTATTCTTCAGCTTCCTCA (*Xho*I)	
NPM1_189–294_	GST-NPM1_189–294_-U	GCGAATTCAAGGCACCAGTGAAGA (*Eco*RI)	pGEX-6P-1
	GST-NPM1_189–294_-L	GCCTCGAGTTAAAGAGACTTCCTCCA (*Xho*I)	
NPM1_1–188_	GST-NPM1_1–188_-U	GCGAATTCATGGAAGATTCGATGGAC (*Eco*RI)	pGEX-6P-1
	GST-NPM1_1–188_-L	GCCTCGAGTTATTCTTCAGCTTCCTCA (*Xho*I)	
NPM1_118–294_	GST-NPM1_118–294_-U	GCGAATTCGCTGTGGAGGAAGATG (*Eco*RI)	pGEX-6P-1
	GST-NPM1_118–294_-L	GCCTCGAGTTAAAGAGACTTCCTCCA (*Xho*I)	
NPM1	Red-NPM1-U	GCCTCGAGATGGAAGATTCGATGGAC (*Xho*I)	pDsRed-N1
	Red-NPM1-L	GCAAGCTTAAGAGACTTCCTCCAC (*Hin*dIII)	
NPM1(T199A)	NPM1(T199A)-2U	CAGTGAAGAAATCTATACGAGATGCTCCAGCCAAAAATGCACAAAA	p3×Flag-CMV-10/pDsRed-N1
	NPM1(T199A)-2L	CTTTTGTGCATTTTTGGCTGGAGCATCTCGTATAGATTTCTTCACTGG	
NPM1(K230R)	NPM1(K230R)-2U	GGACAAGAATCCTTCAAAAGACAGGAAAAAACTCCTAAA	p3×Flag-CMV-10/pDsRed-N1
	NPM1(K230R)-2L	TTTAGGAGTTTTTTCCTGTCTTTTGAAGGATTCTTGT	
NPM1(K263R)	NPM1(K263R)-2U	GGTGGTTCTCTTCCCAGAGTGGAAGCCAAGT	p3×Flag-CMV-10/pDsRed-N1
	NPM1(K263R)-2L	AACTTGGCTTCCACTCTGGGAAGAGAACCAC	
