# Supplementary material for: Nucleocapsid Interacts with NPM1 and Protects it from Proteolytic Cleavage, Enhancing Cell Survival, and is Involved in PEDV Growth
Source: Sci Rep. 2017 Jan 3;7:39700. doi: 10.1038/srep39700 (PMC5206633; doi:10.1038/srep39700)

Supplemental information for the Manuscript **“Nucleocapsid Interacts with NPM1 and Protects it from Proteolytic Cleavage, Enhancing Cell Survival, and is Involved in PEDV Growth”**

Da Shi[1†], Hongyan Shi[1†], Dongbo Sun[2], Jianfei Chen[1], Xin Zhang[1], Xiaobo Wang[1], Jialin Zhang[1], Zhaoyang Ji[1], Jianbo Liu[1], Liyan Cao[1], Xiangdong Zhu[1], Jing Yuan[1], Hui Dong[1], Xin Wang[1], Tiecheng Chang[1], Ye Liu[1], Li Feng[1]*

[1]National Key Laboratory of Veterinary Biotechnology, Harbin Veterinary Research Institute of the Chinese Academy of Agricultural Sciences, No. 678 Haping Road, Xiangfang District, Harbin 150069, P. R. China

[2]College of Animal Science and Veterinary Medicine, Heilongjiang Bayi Agricultural University, No. 2 Xinyang Road, Sartu District, Daqing 163319, P. R. China.

Figure Legends

Supplemental Figure S1. Expression analysis of the Myc-N, 3×Flag-NPM1/F23/C23 in HEK293T cells using western blotting. (A, B and C) Samples were collected from cells transiently transfected with individual expression plasmids at 48 h posttransfection; 100 μg of protein was loaded into each lane for SDS-PAGE and western blotting using mouse anti-N (A), Myc (B) and Flag (C) mAbs as the primary antibodies.

Supplemental Figure S1

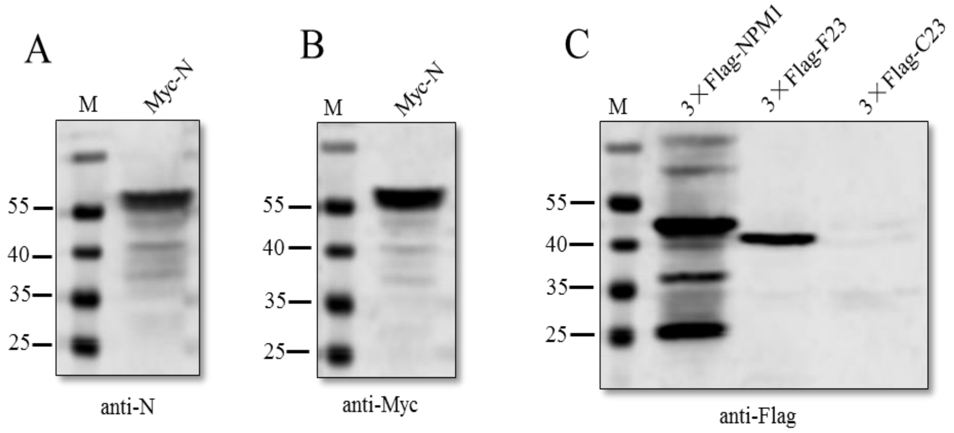

Supplemental Figure S2. PEDV N protein did not interact with fibrillarin or nucleolin.

(A) Co-IP of fibrillarin with N protein. HEK293T cells were co-transfected with the indicated plasmids (+) or empty vectors (–) and the whole-cell lysates (WCL) obtained at 48 hpt were immunoprecipitated with anti-Flag mAb. After separation by SDS-PAGE, proteins were detected by immunoblotting with the indicated antibodies. A 5% aliquot of WCL was also probed to confirm protein expression. The identities of the protein bands are indicated on the right. (B) Co-IP of PEDV N protein with endogenous nucleolin. Mock-infected (M) or PEDV-infected (V) Vero E6 cells were used for immunoprecipitation with anti-N protein mAb and immunoblotted with the indicated antibodies. The identities of the bands are shown on the right.

Supplemental Figure S2

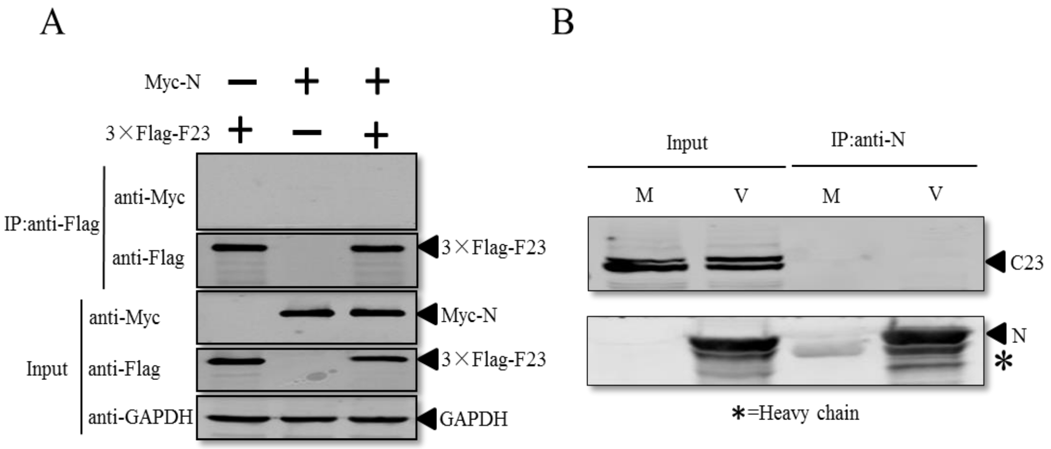

Supplemental Video S3. N protein is imported into the nucleolus independently of NPM1.

The NPM1 fusion protein, used as a marker for the nucleolus, is colored red. The N fusion protein is colored green The nucleolar localization of N in transfected cells was clearly observed at 42–42.5 hpt. As shown, a small amount of N was observed in the nucleolus at 42 hpt. The N protein accumulated continuously in the nucleolus of transfected cells until t=60 min and was exported from the nucleolus at t=61–65 min. Data are representative of one of three independent experiments. Real-time visualization of the kinetics of the nucleolar localization of N protein indicated that the process was rapid, taking only 30 min in total.

Supplemental Figure S4. siRNA treatment does not affect cell viability. Cell viability was detected by CCK-8 assay after transfection with siRNAs in Vero E6 cells for 48 h. Light absorption at 450 nm was recorded and expressed as a percentage of relative cell viability, and the values are represented as the mean ± SD (n=3). Significant differences were assessed by one-way ANOVA. “ns” means no significant difference compared to control, P>0.05.

Supplemental Figure S4

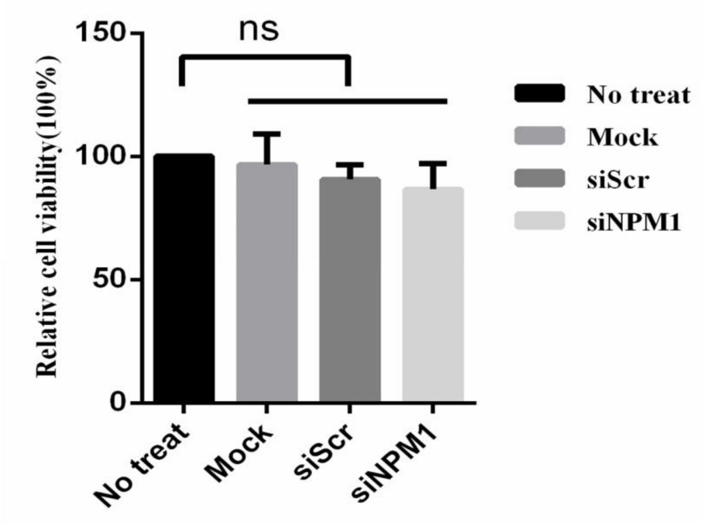

Supplement: Supplementary Information [file srep39700-s1.pdf]
